# Human *Plasmodium knowlesi* Infection Detected by Rapid Diagnostic Tests for Malaria

**DOI:** 10.3201/eid1509.090358

**Published:** 2009-09

**Authors:** Jaap J. van Hellemond, Marijke Rutten, Rob Koelewijn, Anne-Marie Zeeman, Jaco J. Verweij, Pieter J. Wismans, Clemens H. Kocken, Perry J.J. van Genderen

**Affiliations:** Erasmus University Medical Center, Rotterdam, the Netherlands (J.J. van Hellemond); Harbour Hospital and Institute of Tropical Diseases, Rotterdam (M. Rutten, R. Koelewijn, P.J. Wismans, P.J.J. van Genderen); Biomedical Primate Research Centre, Rijswijk, the Netherlands (A.-M. Zeeman, C.H. Klocken); Leiden University Medical Center, Leiden, the Netherlands (J.J. Verweij)

**Keywords:** Plasmodium knowlesi, antigen test, malaria, rapid diagnostics, The Netherlands, dispatch

## Abstract

We describe a PCR-confirmed case of *Plasmodium knowlesi* infection with a high parasitemia level and clinical signs of severe malaria in a migrant worker from Malaysian Borneo in the Netherlands. Investigations showed that commercially available rapid antigen tests for detection of human *Plasmodium* infections can detect *P. knowlesi* infections in humans.

The malaria parasite *Plasmodium knowlesi* naturally occurs in long-tailed and pig-tailed macaques that inhabit forested areas in Southeast Asia. *P*. *knowlesi* can be transmitted from monkeys to humans by the bite of an infected mosquito (*1*), but infection with *P*. *knowlesi* was traditionally regarded as a rare disease, occurring only sporadically in humans. However, recent findings of a large number of infected patients in Malaysian Borneo; other reports of human cases in Thailand, Myanmar, the Philippines, and Singapore; and some reports of *P*. *knowlesi* malaria acquired by travelers to the Malaysian Borneo suggest that *P*. *knowlesi* may be more widespread among humans than previously thought (*2*–*7*).

Unfortunately, microscopic analysis of asexual stages of *P*. *knowlesi* can misidentify these parasites as *P*. *malariae* (*3*,*4*). Unlike *P*. *malariae*, which multiplies every 3 days in the blood and never results in severe infections, *P*. *knowlesi* multiplies daily, and high parasitemia with death in humans can occur (*4*). Therefore, early diagnosis and immediate treatment is warranted. Although PCR and sequencing are used in species confirmation, a more rapid diagnostic test would be a useful tool for delivering prompt and adequate medical treatment. We report a case of imported *P*. *knowlesi* infection in the Netherlands in a migrant worker from Malaysian Borneo. This *P*. *knowlesi* infection was detected by commercially available rapid diagnostic antigen tests for malaria.

## The Case

A 38-year-old man came to the Netherlands in January 2009 to work as a rigger in the harbor of Rotterdam. Since October 2008, he had lived in Kapit, Sarawak, in Borneo and hunted wild animals in the surrounding jungles. One week after arriving in the Netherlands, he came to our hospital with a 5-day history of fever, myalgia, headache, and low back pain. His medical history was uneventful, and he had not experienced any previous malaria attacks.

Physical examination showed a temperature of 40.0°C and remarkable jaundice. Abdominal examination showed no abnormalities. Laboratory investigations showed a moderate anemia (hemoglobin 7.8 mmol/L [reference range 8.5–11.0 mmol/L]), a normal leukocyte count (5.8 × 10^9^/L [reference range 4.3–10 × 10^9^/L]), thrombocytopenia (platelet count 22 × 10^9^/L [reference range 150–400 × 10^9^/L]), an increased level of C-reactive protein (158 mg/L [reference range <10 mg/L]), and liver function abnormalities (serum alanine aminotransferase 199 U/L [reference range <41 U/L]; aspartate aminotransferase 128 U/L [reference range <37 U/L]; lactate dehydrogenase [LDH] 1,059 U/L [reference range <450 U/L]; gamma-glutamyltransferase 183 U/L [reference range <50 U/L]; alkaline phosphatase 285 U/L [reference range <120 U/L]; and total bilirubin 99 μmol/L [reference range <17 μmol/L]). Plasma lactate level was within normal limits.

**Figure 1:**
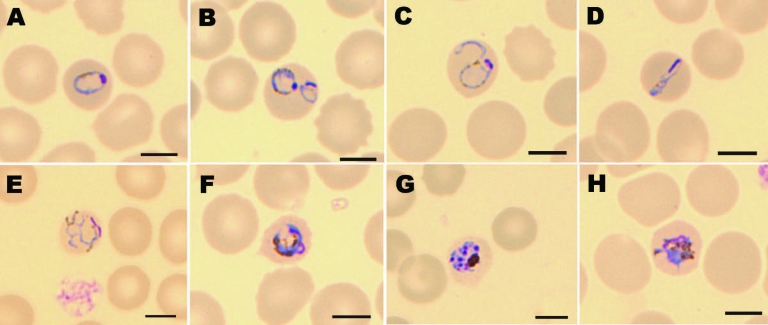
Morphology of *Plasmodium knowlesi* in a Giemsa-stained thin blood smear. Infected erythrocytes were not enlarged, lacked Schuffner stippling, and contained much pigment. Shown are examples of trophozoites (A–F), a schizont (G), and a gametocyte (H). Scale bars = 5 μm.

In a rapid diagnostic test for malaria (BinaxNOW Malaria Test; Binax, Inc., Scarborough, ME, USA), his blood sample was negative for *P*. *falciparum* histidine-rich protein 2 but showed a positive reaction with pan-malarial aldolase antigen, which suggested a non–*P*. *falciparum* infection. Results of quantitative buffy coat analysis were positive for malaria trophozoites, schizonts, and gametocytes. A thin blood film showed parasite density of 2% infected erythrocytes (84,000 trophozoites/μL), schizonts, and gametocytes with an inconclusive morphologic appearance (Figure 1). A *P*. *knowlesi* infection was suspected because of his recent stay in Kapit, Malaysian Borneo. The patient was treated orally with chloroquine, 10 mg/kg, followed by 5 mg/kg after 6, 24, and 48 hours, which resulted in a rapid relief of symptoms and fever. Results of quantitative buffy coat analysis, pan-malarial aldolase antigen reactivity, and thick and thin blood smears were negative within 40 hours after administration of chloroquine.

Subsequently, PCR analysis of blood samples taken at admission was performed to determine the *Plasmodium* species. Human *Plasmodium* species were excluded by using a conventional nested PCR and real-time PCR (*8*,*9*). In addition, PCR analysis was performed on a blood sample by using diagnostic primers for *Plasmodium* small subunit (SSU) rRNA as described (*3*), including genus-specific and species-specific primers. In contrast to the method described by Singh et al. (*3*), nested PCR was not necessary because of high parasitemia and availability of fresh material. Instead, PCRs were performed directly on 2 μL of blood in 25-μL volumes by using the Phusion Blood PCR kit (Finnzymes, Espoo, Finland). Genus-specific primer sets and *P*. *knowlesi*–specific primers generated PCR products, providing evidence that the patient had *P*. *knowlesi* malaria.

**Figure 2:**
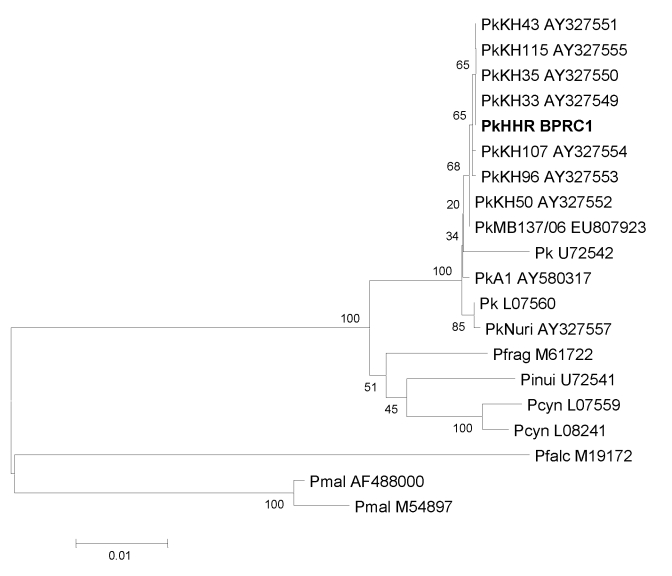
Phylogenetic tree constructed according to the neighbor-joining method based on A-type small subunit RNA sequences of several *Plasmodium* species (GenBank accession numbers are indicated). The sequence of the clinical isolate PkHHR-BPRC1 (in **boldface**) (GenBank accession no. FJ804768) clusters with all other *P. knowlesi* strains (indicated by Pk isolate numbers). Pfrag, *P*. *fragile*; Pinui, *P*. *inui*; Pcyn, *P*. *cynomolgi;* Pfalc, *P*. *falciparum;* Pmal, *P*. *malariae.* Scale bar indicates nucleotide substitutions per site.

To confirm the PCR result, we sequenced the cloned amplification product generated with primers rPLU1 and rPLU5. Sequences were compared with known *Plasmodium* A-type SSU rRNA sequences by using the neighbor-joining method (Figure 2). The sequence of the clinical isolate PkHHR-BPRC1 (GenBank accession no. FJ804768) clustered strongly with *P*. *knowlesi* A-type SSU RNA sequences, confirming that the patient was infected with the *P. knowlesi* parasite.

## Conclusions

Imported malaria is relatively rare in industrialized countries. Obtaining a correct diagnosis of malaria may be troublesome in centers where laboratory staff are less skilled in the proper identification and quantification of causative *Plasmodium* species, as may occur in countries in which malaria is not endemic. These centers often use commercially available rapid diagnostic tests to diagnose malaria. In contrast to our case, Bronner et al. reported that the BinaxNOW Malaria test did not detect a *P*. *knowlesi* infection in a traveler from Sweden who had a *P*. *knowlesi* infection acquired in Malaysian Borneo (*2*). Low parasitemia (0.1%) in this patient may have caused the lack of reactivity with the pan-malarial antigen aldolase (*2*).

We evaluated the BinaxNOW Malaria and the OptiMAL Rapid Malaria (Diamed, Cressier, Switzerland) tests for detection of *P*. *knowlesi* in human blood by analysis of consecutive blood samples taken after admission. These samples were stored at –20°C for 2 weeks until tests were performed (Table). The blood sample taken on admission (2% infected erythrocytes) did not react with *P*. *falciparum*–specific antibody against histidine-rich protein 2, but reacted with the pan-malarial antigen aldolase in the BinaxNOW Malaria Test. This sample also showed a positive result in the *P*. *falciparum*–specific LDH and pan-malarial LDH in the OptiMAL Rapid Malaria test, confirming the cross-reactivity of *P*. *knowlesi* LDH with monoclonal antibody 17E4 against *P*. *falciparum* LDH, as shown by McCutchan et al. (*10*). This antibody is also used in the OptiMAL Rapid Malaria test (Diamed, pers. comm.). Therefore, a positive test result for the *P*. *falciparum* LDH in the OptiMAL Rapid Malaria test is not specific for *P*. *falciparum* because it can also be caused by a *P*. *knowlesi* infection. The positive result for LDH and aldolase in either test became negative after treatment (Table), which indicates rapid clearance of parasites after treatment. Results of our comparative study suggest that the OptiMAL Rapid Malaria test may be able to detect lower levels of *P*. *knowlesi* parasitemia than the BinaxNOW Malaria test.

**Table:** Evaluation of 2 commercially available rapid diagnostic antigen tests used for detection of Plasmodium knowlesi*

Time after admission, h	*P. knowlesi* parasitemia, trophozoites/μL	BinaxNow†			Diamed OptiMAL‡	
		HRP-2	Aldolase		*P. falciparum*–LDH	Pan-malarial LDH
0	84,000	–§	+§		+	+
16	1,587	–§	–§		+	+
24	138	–	–		–	–
40	ND	–	–		–	–

*HRP-2, histidine-rich protein 2; LDH, lactate dehydrogenase; ND, not detectable. All tests were performed on blood samples collected in EDTA and frozen at –20°C for 2 weeks. †Binax, Inc., Scarborough, ME, USA. ‡Diamed, Cressler, Switzerland. §Tests were also performed on freshly collected blood samples; results were identical.

Our results indicate that commercially available rapid diagnostic antigen tests for human *Plasmodium* species can detect *P*. *knowlesi* infections in humans, although infections with a low parasitemia will not be detected. A negative test result does not exclude a *P*. *knowlesi* infection, as it does not exclude infections by other human *Plasmodium* species (*11*).
